# Retrospective Analysis of the Uptake and Timing of Risk-Reducing Salpingo-Oophorectomy in Women With BRCA1/2 Pathogenic Variants

**DOI:** 10.7759/cureus.90772

**Published:** 2025-08-22

**Authors:** Yuri Hasegawa, Shoko Miura, Koh Nagata, Nahoko Komatsu, Ai Nagata, Megumi Matsumoto, Keitaro Matsumoto, Kiyonori Miura

**Affiliations:** 1 Obstetrics and Gynecology, Nagasaki University Graduate School of Biomedical Sciences, Nagasaki, JPN; 2 Surgical Oncology, Nagasaki University Graduate School of Biomedical Sciences, Nagasaki, JPN

**Keywords:** brca1/2 pathogenic variant, decision-making, genetic counseling, hereditary breast and ovarian cancer syndrome (hboc), risk-reducing salpingo-oophorectomy (rrso)

## Abstract

Introduction

Risk-reducing salpingo-oophorectomy (RRSO) can substantially reduce ovarian cancer incidence in women carrying pathogenic *BRCA1* or *BRCA2* variants, which cause hereditary breast and ovarian cancer syndrome. The decision to undergo RRSO or continue surveillance is influenced by personal background and psychosocial factors, but the process in Japan has not been well studied. Even among women who ultimately choose RRSO, some proceed promptly, while others delay. This study aimed to identify factors associated with the decision to undergo RRSO and to examine the timing of that decision, specifically whether it occurred within 365 days of genetic testing.

Methods

We retrospectively reviewed records of patients who either (1) had breast cancer and were found to carry a *BRCA1/2* pathogenic variant, or (2) had a family history and were confirmed as carriers. *BRCA1/2* testing was performed using next-generation sequencing covering all coding regions and exon-intron boundaries, with confirmatory Sanger sequencing. Data collected included age at consultation, parity, history of breast cancer and treatment, genetic testing date and result, RRSO date, insurance coverage, pathology findings, and reasons for surveillance. Multivariable logistic regression assessed factors associated with undergoing RRSO and with undergoing RRSO within 365 days. Kaplan-Meier analysis and Cox regression were used to evaluate time-to-RRSO. Statistical significance was set at p < 0.05.

Results

A total of 70 patients were analyzed (mean age 47.8 years (SD 13.1); 55 (78.6%)) had breast cancer. Pathogenic variants were in *BRCA1* (n=26, 37.1%) or *BRCA2* (n=44, 62.9%). A total of 32 patients (45.7%) underwent RRSO. Multivariable analysis showed age >45 years (p = 0.0202, OR: 4.00, 95% CI: 1.24-12.9) and breast cancer history (p = 0.0247, OR: 7.68, 95% CI: 1.30-45.4) were significantly associated with RRSO. Among those undergoing RRSO within 365 days, *BRCA1* carriers were more likely than *BRCA2* carriers to have early surgery (p = 0.0471, OR: 6.81, 95% CI: 1.02-45.3). Kaplan-Meier analysis showed shorter median time to RRSO for *BRCA1* carriers (565 days) vs. *BRCA2* carriers (1,015 days); Cox regression findings were consistent.

Conclusion

Age and breast cancer history were important factors in the decision to undergo RRSO. Earlier RRSO among *BRCA1* carriers suggests that genetic counseling effectively conveys their higher and earlier ovarian cancer risk. These results highlight the importance of individualized counseling that considers each patient’s background, supporting informed decisions about both RRSO uptake and timing.

## Introduction

Hereditary breast and ovarian cancer syndrome (HBOC) predisposes women carrying pathogenic variants of the *BRCA1* and *BRCA2* genes to a significantly increased risk of developing breast, ovarian, and fallopian tube cancer [1]. There are currently no established effective surveillance methods for the early detection of ovarian cancer [2].

The risk of ovarian cancer in *BRCA1/2* pathogenic variant carriers can be significantly reduced by risk-reducing salpingo-oophorectomy (RRSO), with a reported hazard ratio (HR) of 0.06, compared with that in non-carriers who did not undergo the procedure [3]. The National Comprehensive Cancer Network (NCCN) guidelines [4] recommend RRSO for ovarian cancer risk reduction by age 35-40 years for BRCA1 mutation carriers, and by age 40-45 years for *BRCA2* variant carriers. Recent studies have also demonstrated that RRSO reduces the risk of breast cancer [5].

Despite these recommendations, whether a woman with a *BRCA1/2* pathogenic variant opts for RRSO or surveillance varies by individual. This variability is influenced by personal background factors and psychosocial considerations [6]. However, little is known about the factors that influence such decisions in Japan. Understanding the factors that influence the decision to undergo RRSO is crucial for delivering appropriate genetic counseling to *BRCA1/2* variant carriers.

Even among women who ultimately choose RRSO, some make the decision to undergo RRSO promptly, while others delay surgery until after a period of surveillance. To date, no studies have explored the factors associated with the delayed decision-making regarding RRSO.

In this study, we aimed to investigate the factors influencing the decision to undergo RRSO among women with *BRCA1/2* pathogenic variants who received genetic counseling at our institution. In addition, as an integral secondary objective, we examined factors associated with the timing of this decision, specifically whether RRSO was performed within 365 days of genetic testing. This dual focus was intended to capture not only whether patients chose to undergo RRSO, but also how promptly the decision was made, given that delaying surgery beyond guideline-recommended ages may have clinical implications for cancer risk.

## Materials and methods

Study participants

**Figure 1:**
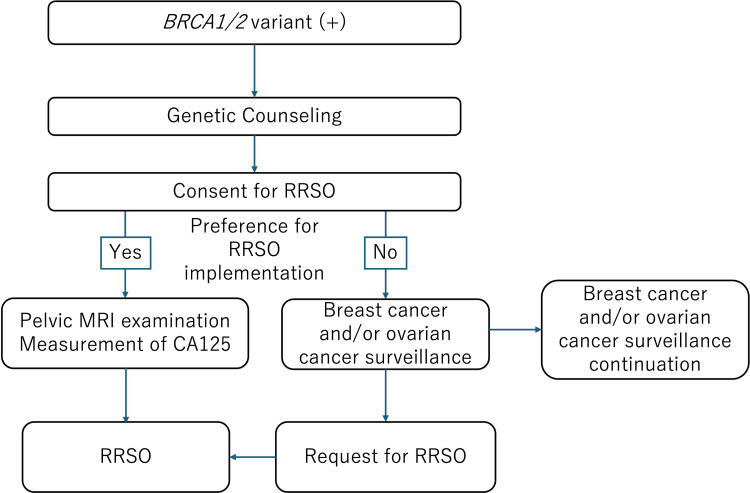
The protocol used for decision-making This flowchart illustrates the clinical protocol followed for *BRCA1/2* pathogenic variant carriers considering RRSO. All individuals identified as *BRCA1/2* positive undergo genetic counseling. Based on their preference, those who opt for RRSO receive a pelvic MRI and serum CA125 measurement as part of the preoperative assessment. Upon expressing a desire to proceed, they are provided with information and subsequently consent to the procedure. RRSO is then performed. Individuals who decline RRSO continue with regular breast and/or ovarian cancer surveillance.

This retrospective study included patients who, between January 1, 2013, and March 31, 2025, either (1) were diagnosed with breast cancer and subsequently underwent genetic testing that identified a pathogenic variant in *BRCA1* or *BRCA2*, or (2) had a family history of a *BRCA1/2* pathogenic variant and were themselves found to carry a pathogenic variant following testing. *BRCA1/2* genetic testing was outsourced to SRL, Inc. (Tokyo, Japan) and performed by Myriad Genetics (Salt Lake City, UT, USA) using a next-generation sequencing platform covering the entire coding regions and exon-intron boundaries of both genes. Pathogenic or likely pathogenic variants identified by next-generation sequencing were confirmed by Sanger sequencing. All patients received genetic counseling at our institution and were informed of the benefits and drawbacks of both RRSO and surveillance. Based on this counseling, they chose either RRSO or continued surveillance. Surveillance comprised transvaginal ultrasonography and measurement of serum CA125 levels every 4-6 months. The protocol used for decision-making is illustrated in Figure 1.

Genetic counseling

All patients identified as carrying a *BRCA1/2* pathogenic variant were referred to the Division of Clinical Genetics at Nagasaki University Hospital, where they received counseling from a multidisciplinary team comprising board-certified specialists in obstetrics and gynecology, breast surgery, and certified genetic counselors. Genetic counseling was conducted over multiple sessions and included a detailed explanation of the advantages and limitations of both RRSO and surveillance. The decision to proceed with RRSO or continue surveillance was made respecting the patient's preferences.

RRSO and pathological diagnosis

For patients who chose to undergo RRSO, preoperative assessment included pelvic magnetic resonance imaging (MRI) and tumor marker (CA125) testing to confirm the absence of abnormalities. The RRSO procedure was performed according to established and recommended surgical guidelines [7,8]. Pathological examination was conducted by board-certified pathologists using the Sectioning and Extensively Examining the FIMbriated End (SEE-FIM) protocol [9].

Data collection and variables

The following data were retrospectively collected from medical records: age at the time of counseling; parity; history of breast cancer, including treatment details if applicable; date of genetic testing; results of *BRCA* testing; date of RRSO, if performed; whether RRSO was covered by insurance or self-paid; pathological findings from RRSO; and reasons for not undergoing RRSO. Reasons for selecting surveillance over RRSO were also documented. In Japan, RRSO became eligible for insurance coverage in April 2020 for patients diagnosed with HBOC after developing breast cancer [7]. Surgeries performed before March 31, 2020, or prophylactic RRSO in unaffected *BRCA1/2* carriers were considered out-of-pocket procedures.

Statistical analysis

To evaluate the factors associated with the decision to undergo RRSO, logistic regression analysis was performed using the following variables: age >45 years, parity, facility where *BRCA* testing was conducted, type of *BRCA* pathogenic variant (*BRCA1* or *BRCA2*), and history of breast cancer.

Among patients who underwent RRSO, additional logistic regression was conducted to identify factors associated with undergoing RRSO within 365 days of *BRCA* testing. The following variables were included: age >45 years, parity, *BRCA* testing facility, *BRCA* variant type, history of breast cancer, and whether the RRSO was covered by insurance. A p-value of less than 0.05 was considered statistically significant. Multicollinearity among the independent variables was assessed using variance inflation factors (VIFs), and all VIFs were below 3.0, indicating no evidence of multicollinearity.

We analyzed the time from genetic testing to RRSO using Kaplan-Meier methods and Cox proportional hazards regression to leverage the time-to-event nature of the outcome. Participants without RRSO were right-censored at their last follow-up date. Covariates included medical history (breast cancer vs. unaffected), hospital (in-hospital vs. outside), *BRCA* status (*BRCA1* vs. *BRCA2*), insurance status (insured vs. self-pay), parity (parous vs. nulliparous), and age >45 years (yes/no). Hazard ratios (HRs) with 95% confidence intervals (CIs) were reported. We also fit a logistic regression model using a 365-day cut-off.

Additionally, to assess the impact of sample size on our multivariable logistic regression of RRSO uptake, we performed a post hoc power calculation using a Wald-test approximation (two-sided α=0.05) based on the observed coefficients and standard errors. The estimated power was 48.5% for age >45 years and 60.6% for breast cancer history (unaffected vs. breast cancer), whereas other covariates showed low power (6.3-18.2%), indicating limited sensitivity to detect weaker effects.

All statistical analyses were performed using JMP Pro 16 (version 16.2.0; SAS Institute Inc., Cary, NC, USA)

Ethical approval

This study was approved by the Ethics Committee of Nagasaki University Hospital (approval number: 25061969). As this was a retrospective review of existing medical records, the requirement for written informed consent was waived by the committee in accordance with national and institutional guidelines for clinical research.

## Results

Patient characteristics

The characteristics of the study population are summarized in Table 1. A total of 70 patients were included. The mean age was 47.8 years (standard deviation [SD]: 13.1), and 51 patients (72.9%) were parous. A history of breast cancer was observed in 55 patients (78.6%), and 47 patients (67.1%) underwent *BRCA1/2* genetic testing at our institution. Pathogenic variants were identified in *BRCA1* in 26 patients (37.1%) and in* BRCA2* in 44 patients (62.9%).

**Table 1:** Patient characteristics (n = 70) RRSO: risk-reducing salpingo-oophorectomy, SD: standard deviation.

Characteristic	Average (SD) or Number (%)
Age	47.8±13.1
History of delivery	
One or more	51 (72.9%)
None	19 (27.1%)
Medical history	
Breast cancer	55 (78.6%)
No onset	15 (21.4%)
*BRCA1/2* gene testing facility	
Our hospital	47 (67.1%)
Others	23 (32.9%)
Genetic variant	
BRCA1	26 (37.1%)
BRCA2	44 (62.9%)
RRSO performed	
Yes	32 (45.7%)
No	38 (54.3%)
Period from genetic testing to RRSO (if performed) (days)(n=32)	405.9±241.8
RRSO covered by insurance (n=32)	
Yes	29 (90.6%)
No	3 (9.4%)

**Figure 2:**
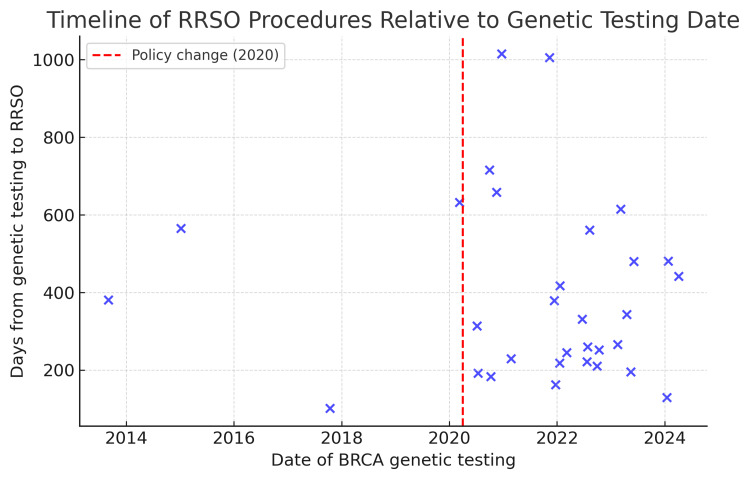
Timeline of RRSO procedures relative to BRCA genetic testing date. Each blue dot represents an individual patient who underwent risk-reducing salpingo-oophorectomy (RRSO), plotted according to the date of *BRCA* genetic testing on the x-axis and the number of days from genetic testing to RRSO on the y-axis. The red dashed vertical line indicates the policy change implemented in April 2020, when insurance coverage for RRSO became available in Japan [7].

Of the 70 patients, 32 (45.7%) underwent RRSO. Among these 32 patients, one case (3.1%) was diagnosed with stage IIA serous carcinoma originating from the surface of the resected ovary. The mean interval from the date of genetic testing to the date of RRSO was 405.9 days (SD: 241.8). RRSO was performed under public health insurance coverage in 29 of the 32 patients (90.6%). A timeline plot illustrating the interval between *BRCA* genetic testing and RRSO is shown in Figure 2. Each point represents an individual who underwent RRSO, with the x-axis indicating the date of *BRCA* genetic testing and the y-axis representing the number of days from testing to surgery. The distribution shows substantial variability in the timing of RRSO, ranging from less than 200 days to more than 2000 days after testing. The red dashed line marks April 2020, when insurance coverage for RRSO was introduced in Japan [7], after which an increase in earlier RRSO procedures is observed.

Factors associated with the decision to undergo RRSO

A logistic regression analysis was performed to evaluate which clinical background factors were associated with the decision to undergo RRSO, including age >45 years, parity, *BRCA* testing facility, *BRCA* variant type, and history of breast cancer. The results are shown in Table 2. Among the five variables assessed, age >45 years (p = 0.0202, odds ratio (OR): 4.00, 95% confidence interval (CI): 1.24-12.9), and a history of breast cancer (p = 0.0247, OR: 7.68, 95% CI: 1.30-45.4) were significantly associated with RRSO uptake. The variance inflation factors (VIFs) for the independent variables ranged from 1.19 to 2.78, indicating no significant multicollinearity in the model.

**Table 2:** Factors associated with the decision to undergo RRSO RRSO: risk-reducing salpingo-oophorectomy, NS: not significant, CI: confidence interval * P-values were determined by logistic regression analysis.

Factors	Odds ratio (95% CI)	P-value*
Age (years) >45	4.00 (1.24-12.9)	0.0202
History of delivery	1.05 (0.27-4.03)	NS
*BRCA1/2* gene testing performed at our hospital	2.00 (0.59-6.79)	NS
*BRCA1* pathogenic variant present	2.06 (0.63-6.72)	NS
History of breast cancer	7.68 (1.30-45.4)	0.0247

Factors associated with undergoing RRSO within 365 Days of *BRCA* testing

**Figure 3:**
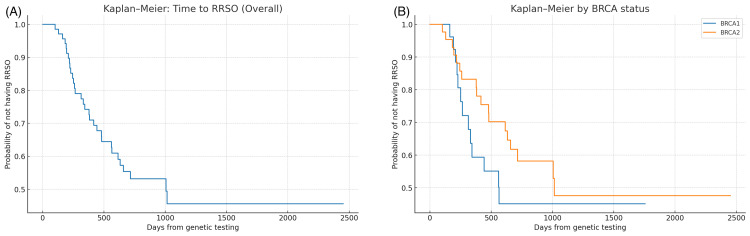
Kaplan–Meier curves for time from genetic testing to risk-reducing salpingo-oophorectomy (RRSO). (A) Overall survival curve showing the probability of not undergoing RRSO over time since genetic testing. The median time to RRSO was 1006 days (95% CI, not calculated due to stepwise estimation limits). (B) Stratified by *BRCA* status, the median time to RRSO was shorter for *BRCA1* carriers (565 days) than for *BRCA2* carriers (1,015 days). Participants without RRSO were censored at their last follow-up date. Shaded areas represent 95% confidence intervals.

Kaplan-Meier analysis of the time from *BRCA1/2* genetic testing to RRSO showed a median time of 1,006 days overall, 565 days for *BRCA1* carriers, and 1,015 days for *BRCA2* carriers (Figure 3). Participants without RRSO were censored at their last follow-up date.

In multivariable Cox proportional hazards regression adjusting for age >45 years, parity, *BRCA* testing facility, *BRCA* variant type, history of breast cancer, and insurance coverage status, being unaffected (vs. having a history of breast cancer) was significantly associated with a lower hazard of undergoing RRSO (HR: 0.21, 95% CI: 0.05-0.91). Age >45 years (HR: 1.96, 95% CI: 0.86-4.46) and self-pay insurance (HR: 2.57, 95% CI: 0.67-9.78) were associated with higher hazards, whereas carrying a *BRCA2* pathogenic variant (vs. *BRCA1*) showed a lower hazard (HR: 0.64, 95% CI: 0.34-1.19), although these did not reach statistical significance (Table 3).

**Table 3:** Multivariable Cox proportional hazards regression for time from genetic testing to RRSO. Participants without RRSO were censored at their last follow-up date. Covariates included medical history, hospital, *BRCA* status, insurance status, parity, and age >45 years. p-values are from Wald tests. HR, hazard ratio; CI, confidence interval; NS, not significant

Variable	HR	95% CI lower	95% CI upper	P-value
Age >45 years	1.96	0.86	4.46	NS
Medical history: unaffected (vs. breast cancer)	0.21	0.05	0.91	NS
Hospital: outside (vs. in-hospital)	0.61	0.28	1.33	NS
BRCA2 (vs. BRCA1)	0.64	0.34	1.19	NS
Insurance: self-pay (vs. insured)	2.57	0.67	9.78	NS
Parity: parous (vs. nulliparous)	0.94	0.45	1.97	NS

As a supportive analysis to facilitate comparison with prior studies, logistic regression was also performed using an outcome of undergoing RRSO within 365 days of *BRCA1/2* genetic testing (Table 4). The following variables were examined: age >45 years, parity, *BRCA* testing facility, *BRCA *variant type, history of breast cancer, and insurance coverage status of the RRSO. Among the six variables assessed, carrying a *BRCA1* pathogenic variant (as opposed to *BRCA2*) was significantly associated with undergoing RRSO within 365 days (p = 0.0471, OR: 6.81, 95% CI: 1.02-45.3). The variance inflation factors (VIFs) for the independent variables ranged from 1.11 to 2.78, indicating no significant multicollinearity in the model.

**Table 4:** Factors associated with undergoing RRSO within 365 Days of BRCA testing RRSO: risk-reducing salpingo-oophorectomy, NS: not significant, CI: confidence interval. * P-values were determined by logistic regression analysis.

Factors	Odds ratio (95% CI)	P-value*
Age (years) >45	0.59 (0.08-4.29)	NS
History of delivery	4.93 (0.48-50.4)	NS
*BRCA1/2* gene testing performed at our hospital	1.44 (0.23-8.71)	NS
*BRCA1* pathogenic variant present	6.81 (1.02-45.3)	0.0471
History of breast cancer	1.78 (0.05-58.2)	NS
RRSO covered by insurance	1.31 (0.07-25.9)	NS

Reasons for not undergoing RRSO

The reasons for not undergoing RRSO are summarized in Table 5. Among the 38 patients who did not undergo RRSO, the most frequently reported reason for choosing surveillance was being asymptomatic and/or young (n = 17, 44.7%). The second most common reason was ongoing treatment for breast cancer (n = 13, 34.2%). Other reasons included occupational or environmental factors (n = 4, 10.5%). In four patients (10.5%), no specific reason was documented.

**Table 5:** Reasons for not undergoing RRSO (n=38) RRSO: risk-reducing salpingo-oophorectomy

Reasons	Number (%)
No cancer onset and/or young age	17 (44.7)
Currently undergoing treatment for breast cancer	13 (34.2)
Work or family environment	4 (10.5)
No reason	4 (10.5)

## Discussion

This study provides important insights into the patient-related factors that influence the decision to undergo RRSO among carriers of *BRCA1/2* pathogenic variants. In addition, it offers a novel perspective by examining the factors associated with the delayed implementation of RRSO among those who ultimately opted for the procedure.

Patient characteristics

Previous reports from Japan have indicated that approximately 30% of patients diagnosed with HBOC undergo RRSO [10]. In contrast, our study demonstrated a higher RRSO uptake rate of 45.7%. This discrepancy may be attributed to the composition of our study population, which consisted primarily of patients with a personal history of breast cancer or a strong family history of HBOC who were confirmed as carrying a *BRCA1/2* pathogenic variant. The high rate of RRSO implementation observed in this study may also be partly attributed to the fact that over 90% of RRSO procedures were covered by public health insurance. Matsumoto et al. [11] examined the rates of RRSO before and after insurance coverage was introduced in Japan and reported a significantly higher implementation rate following coverage. Their findings are consistent with the results of the present study. Furthermore, since the report by Nomura et al. [10] analyzed cases prior to the inclusion of RRSO in the national health insurance coverage in Japan, this may partly account for the higher rate of RRSO in our cohort. In our cohort, 37.1% carried a *BRCA1* variant and 62.9% carried a *BRCA2* variant. Compared with the distribution reported by Arai et al. [12] (*BRCA1*: 40%, *BRCA2*: 18%), the proportion of *BRCA2* carriers was greater in our study.

Factors associated with the decision to undergo RRSO

Our logistic regression analysis revealed that age >45 years and a history of breast cancer were significantly associated with the decision to undergo RRSO. This finding is consistent with previous reports by Mai et al. [6], who demonstrated that older age, breast cancer history, and postmenopausal status were associated with a greater likelihood of choosing RRSO over surveillance. Conversely, patients who opted for surveillance were more likely to express concerns about surgical complications and menopausal symptoms. In our study, older age was a significant factor in the decision to undergo RRSO. This study included patients who were diagnosed with pathogenic *BRCA1/2* variants either after developing breast cancer or based on family history, and therefore, selection bias in the dataset is inevitable. However, a history of breast cancer is likely a significant factor influencing the decision to undergo RRSO because of the heightened anxiety regarding future cancer development.

Factors associated with the timing of RRSO after *BRCA* testing

Among the six clinical background factors analyzed (age >45 years, parity, *BRCA* testing facility, *BRCA* variant type, breast cancer history, and insurance coverage eligibility), only *BRCA* variant type was significantly associated with undergoing RRSO within 365 days of genetic testing. Specifically, patients carrying a *BRCA1* pathogenic variant were more likely to undergo RRSO within one year compared with those carrying a *BRCA2* variant. This finding suggests that, through genetic counseling, patients may become aware that *BRCA1* carriers face a higher risk of developing ovarian cancer at an earlier age.

Kaplan-Meier analysis further supported this observation, demonstrating that the median time from genetic testing to RRSO was markedly shorter for *BRCA1* carriers (565 days) than for *BRCA2* carriers (1,015 days). Although in the multivariable Cox proportional hazards model, the hazard ratio for *BRCA2* versus *BRCA1* did not reach statistical significance, the direction of the association was consistent with the 365-day logistic regression, indicating a tendency for *BRCA1* carriers to undergo RRSO earlier. These complementary analyses reinforce the concept that genetic counseling may influence not only the decision to undergo RRSO but also the timing of surgery.

Smith et al. investigated delayed RRSO in women with *BRCA1/2* mutations [13] and reported that among 638 carriers, 306 (48.0%) underwent RRSO. They defined delayed RRSO as surgery performed after age 40 in *BRCA1* carriers or after age 45 in *BRCA2* carriers. Patients in the delayed group were more likely to be younger and have a history of cancer at the time of decision-making. Although the study setting of Smith et al. differs from ours, our findings underscore the importance of delivering accurate, age-specific information during genetic counseling - particularly to *BRCA1* carriers - and ensuring timely follow-up to support appropriate surgical decision-making.

Reasons for not undergoing RRSO

Among patients who declined RRSO and chose surveillance, the most commonly cited reasons were being asymptomatic and/or young and undergoing active treatment for breast cancer. Smith et al. [13] identified similar factors contributing to delayed RRSO, including delayed identification of *BRCA* mutations, concerns about menopausal symptoms, and ongoing cancer treatment. In our study, lack of remission from the primary breast cancer was a key factor in the decision to postpone RRSO. These findings highlight the importance of integrating the status of the primary disease into the genetic counseling process when determining the appropriate timing for RRSO. For young and asymptomatic carriers, continued surveillance and ongoing counseling are essential. Notably, recent studies have shown that RRSO may also reduce the risk of breast cancer [3,14,15], which should be included as part of the comprehensive counseling content. Maintaining regular contact with patients and reassessing their readiness for RRSO over time is vital to support informed and timely decision-making.

Strengths and limitations

A key strength of our study is that we not only identified patient-related factors associated with the decision to undergo RRSO but also examined how these factors influenced the timing of surgery. Furthermore, because all genetic counseling was conducted at a single institution, the consistency and quality of counseling were maintained across all participants.

However, this study has several limitations. First, the sample size was relatively small, which may limit the statistical power to detect weaker associations and reduce the generalizability of the findings. Our post hoc power analysis indicated modest power for age >45 years (48.5%) and breast cancer history (60.6%), with low power for other covariates (6.3-18.2%), underscoring the need for larger cohorts. Second, as a retrospective single-center study, there may be unmeasured confounding factors influencing decision-making and timing that were not captured in our dataset. Because the cohort consisted of individuals referred to a single tertiary center for genetic counseling, referral/selection bias is possible, and the cohort may not fully represent the broader *BRCA1/2 *carrier population in Japan. Future multicenter studies with larger and more diverse populations are warranted to validate and extend these findings.

## Conclusions

Our study suggests that active breast cancer treatment may be a key reason for deferring RRSO, further underscoring the essential role of tailored counseling and longitudinal support for *BRCA1/2* mutation carriers. The significance of this study lies in its investigation of the factors associated with a delay of more than one year between the diagnosis of a *BRCA1/2* pathogenic variant and the performance of RRSO.

Furthermore, it provides a detailed analysis of the reasons underlying such delays, which adds substantial value to the current understanding of clinical decision-making in this context. However, these conclusions should be interpreted with caution given the relatively small sample size and the retrospective single-center design, and further multicenter studies with larger cohorts are warranted to validate and generalize these findings.
